# Travel vaccination in senior travelers: current evidence, challenges, and prevention

**DOI:** 10.1186/s40794-026-00315-1

**Published:** 2026-09-14

**Authors:** Andrea Rossanese, Alberto Tomasi

**Affiliations:** 1 Department of Infectious-Tropical Diseases and Microbiology, IRCCS “Sacro Cuore-Don Calabria”, Negrar di Valpolicella, Viale Rizzardi, 4, 37024 Verona, Italy; 2 Italian Society of Travel and Migration Medicine (SIMVIM), Livorno, Italy

**Keywords:** Senior travelers, Travel medicine, Vaccination, Older adults, Pre-travel consultation, Immunosenescence, Yellow fever, Travel vaccines

## Abstract

The substantial number of older adults undertaking international travel has increased the relevance of pre-travel prevention in this population. However, vaccination strategies in this group require consideration of immune aging, baseline status, medication use, and travel-related exposure. This narrative review summarizes the current evidence on vaccination in senior travelers, with particular attention to the vaccines most relevant to this population, the differences in the strength of evidence across vaccines, and the clinical factors that should guide pre-travel decision-making. Routine vaccines, including influenza, COVID-19, pneumococcal, respiratory syncytial virus, herpes zoster, and tetanus-diphtheria vaccines, constitute an essential part of pre-travel assessment, whereas travel-specific vaccines, such as hepatitis A, hepatitis B, typhoid, rabies, Japanese encephalitis, tick-borne encephalitis, and cholera, should be considered according to the destination, itinerary, expected exposure, and time before departure. Yellow fever vaccination represents a particular clinical challenge because advancing age is associated with a greater risk of serious vaccine-associated adverse events, making individualized risk‒benefit assessment essential. Recently introduced dengue and chikungunya vaccines should also be considered on an individualized basis, taking into account exposure risk, potential benefits, comorbidities, and the current limitations of the available evidence. The review also highlights the importance of integrating vaccination with non-vaccine preventive strategies, including food and water precautions, vector avoidance, and hygiene measures. Although the evidence base has expanded, important uncertainties remain, particularly because age-specific data are limited and frail older adults remain underrepresented in clinical studies. Overall, pre-travel care for senior travelers should be comprehensive, exposure-based, and individualized according to the clinical profile and travel characteristics of each traveler.

## Introduction

Older adults represent a substantial and clinically relevant group within contemporary travel medicine. In Europe, tourists aged 65 years or older accounted for nearly one in four tourism nights for private purposes among EU residents in 2019, while people aged 55 years or older accounted for 41% of such nights [1]. In the United States, a 2025 AARP survey reported that 70% of adults aged 50 years or older planned to travel in 2025, including 68% of those aged over 60 years, and 44% were considering international travel [2]. In travel medicine settings, the Spanish +Redivi network identified 1,230 of 29,573 tourist travelers seeking post-travel care between 2011 and 2023 aged ≥ 60 years [3], while the Dutch ELDEST prospective cohort followed 477 travelers aged ≥ 60 years after pre-travel consultation, with a median age of 66 years and a median travel duration of 19 days [4]. Adults aged 65 years or older often combine destination-specific infectious exposure with age-related factors that may alter both susceptibility to infection and the clinical consequences of travel-associated illness [5]. Accordingly, although individualized pre-travel assessment is a general principle of travel medicine, it is particularly important in older travelers because this group may include both healthy, active individuals and travelers with substantial comorbidity, polypharmacy, or reduced physiological reserve [6].

Vaccination remains a central component of pre-travel prevention in older adults [7]. However, age-related immune changes may reduce vaccine responses, shorten protection, and affect tolerability [8]. Consequently, vaccine performance and the balance between expected benefits and potential risk may differ from those observed in younger travelers [9]. For some vaccines, older age may also be associated with a greater risk of adverse events, reinforcing the need for careful evaluation of contraindications and individualized clinical judgment during pre-travel consultation [3].

At the same time, vaccination alone does not fully address the preventive needs of senior travelers [8]. Because travel-related risk reflects both host vulnerability and exposure context, pre-travel care should also incorporate counseling on food and water precautions, vector avoidance, personal protective measures, and the optimization of chronic comorbidities before departure [5]. Pre-travel consultation should therefore integrate routine immunization status, destination-specific vaccine indications, and non-vaccine preventive strategies within an overall risk assessment [10].

Despite the relevance of older adults undertaking international travel, the evidence base supporting vaccination in this population remains limited and heterogeneous [8]. Much of the available guidance continues to rely on data extrapolated from younger adults or from broader populations not specifically designed to evaluate travelers aged 65 years or older [11]. As a result, important uncertainties persist regarding vaccine immunogenicity, effectiveness, safety, and optimal use in senior travelers [12]. This narrative review synthesizes the current evidence on vaccination in senior travelers, focusing on the vaccines most relevant to this population, recent developments in respiratory, pneumococcal, dengue, and chikungunya vaccination, the differences in the strength of the available evidence, and the main evidence gaps that continue to limit more precise guidance.

## Defining the senior traveler at risk

Although age thresholds remain operationally useful, chronological age alone is an imperfect marker of travel-related vulnerability, since the higher risk of severe imported disease in older travelers is driven largely by the greater prevalence of underlying comorbidities rather than by age alone [3]. A study from 2021 reported that travelers aged ≥ 60 years were generally fit, with a median Charlson Comorbidity Index score of 0, although 16% reported polypharmacy, illustrating the coexistence of preserved functional status and clinically relevant risk factors in this population [4]. In addition, physiological changes associated with aging, including reductions in cardiac output, maximal heart rate, maximal oxygen consumption, muscle mass, and cognitive reserve, may reduce tolerance to the physical and environmental demands of travel, even in the absence of severe chronic disease [5]. At the immunological level, immunosenescence may weaken responses to both infection and vaccination, contributing to less predictable protection in older adults [8, 13].

Risk is further shaped by the high burden of baseline medical conditions in this population. Approximately half of elderly travelers present with at least one pre-existing comorbidity or elevated risk factor during pre-travel screening, while in the broader older population chronic conditions, polypharmacy, and mobility impairment are highly prevalent [5, 14]. This burden is clinically relevant because travelers with pre-existing medical conditions have been reported to have more than twice the risk of travel-related illness compared with healthy travelers [15]. Frequent conditions such as hypertension, diabetes, and chronic pulmonary disease may complicate both the presentation and management of travel-associated illness, while reduced functional reserve may increase susceptibility to altitude illness, heat-related disorders, dehydration, and falls in unfamiliar environments [3, 8, 12].

Importantly, adults aged 65 years or older do not constitute a homogeneous group. They range from frail individuals with cognitive impairment, reduced mobility, and substantial dependence on healthcare systems to healthy and active retirees undertaking vigorous outdoor activities or adventure travel [3, 5]. Travel style and budget are also variable, and a relevant minority undertake low-budget travel to destinations with limited medical infrastructure and exposure to infections such as yellow fever and malaria [6, 16]. This heterogeneity supports assessing the planned journey in relation to each traveler’s health status and functional capacity.

## PRE-travel risk assessment and vaccine response in senior travelers

**Fig. 1:**
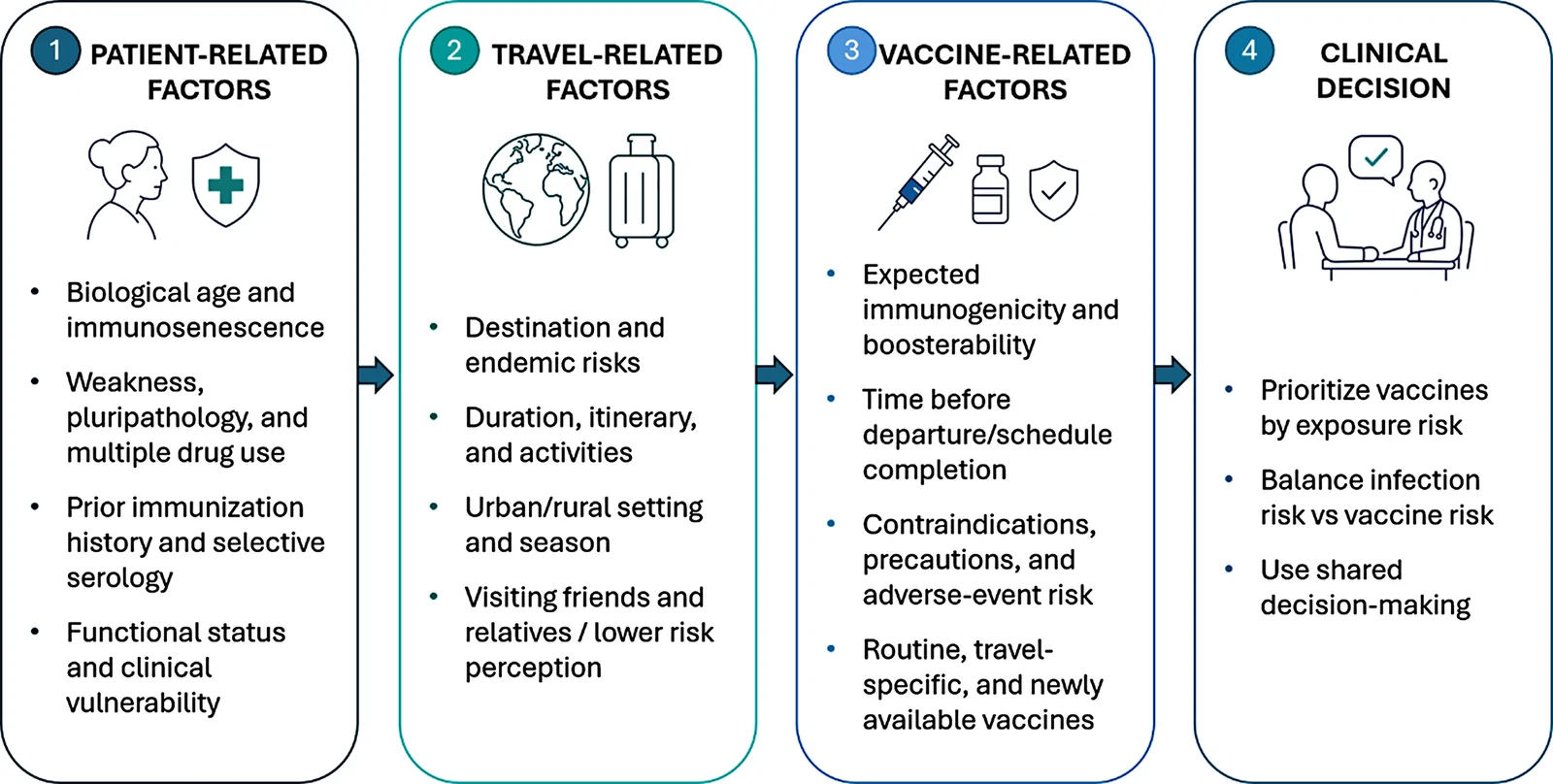
Comprehensive pre-travel health assessment in older adults

Pre-travel assessment in older adults should go beyond a simple review of vaccination records and integrate exposure risk, prior immunity, time before departure, and vaccine-specific safety considerations (Fig. 1) [8].

Immunosenescence, defined as the gradual age-related deterioration of the immune system, is a key biological determinant of the vaccine response in older adults [17]. It affects both innate and adaptive immunity, leading to impaired T-cell and B-cell function, weaker cellular responses, and lower antibody titres after vaccination [18]. For example, lower seroprotection has been reported in older adults after hepatitis A vaccination following the first dose (65% vs. 100%) [19] and after hepatitis B vaccination (64.8% in adults aged ≥ 65 years vs. 98.6% in those aged 20–24 years) [8]. This effect is compounded by the high prevalence of chronic cardiovascular, pulmonary, and metabolic conditions [20]. Together, immunosenescence, chronic low-grade inflammation, and baseline comorbidities increase the risk of severe travel-related infections and support the need for individualized preventive strategies [21].

As in all pre-travel consultations, prior immunization history should therefore be reviewed actively, since undocumented protection cannot be assumed in travelers with uncertain vaccination records. Selective serology may be clinically useful in travelers with uncertain vaccination histories. In a retrospective audit of pre-travel serology for measles, mumps, rubella, varicella zoster virus, hepatitis A, and hepatitis B, 47% of tested individuals had at least one negative serological result, indicating potential susceptibility to one or more vaccine-preventable infections [22]. In older adults, incomplete prior protection appears particularly relevant for routine vaccines such as tetanus-containing vaccines [23]. However, serological testing has important interpretative limitations. Antibody titres are only one component of vaccine-induced immunity, and low or undetectable antibody levels do not necessarily indicate complete loss of immune memory. Memory B-cell responses, T-cell-mediated immunity, and the capacity to mount an anamnestic response after booster vaccination may persist despite waning circulating antibodies [18].

In senior travelers, vaccination priorities should be driven by the planned itinerary and expected exposures, rather than by age alone. This is especially important because older travelers may undertake both short and prolonged journeys to regions such as Africa, Asia, and South America, where preventive priorities differ substantially [3, 23, 24]. A particularly vulnerable subgroup consists of travelers visiting friends and relatives, who often spend more time in rural settings, adopt local dietary and hygienic practices, and show lower risk perception, with correspondingly lower rates of pre-travel health-seeking behavior and vaccine uptake [25].

Time before departure is another critical variable since it determines whether vaccine schedules can be completed in time to provide meaningful protection. This is particularly relevant in older adults, in whom initial responses after a first dose may be weaker, making partial schedules less reliable when travel is imminent [8]. These findings support timely pre-travel assessment, as for example, the initial age-related difference in hepatitis A vaccine response appears to be attenuated after booster vaccination, with seroprotection reaching 97% in older adults and 100% in younger adults [19]. Risk‒benefit assessment should also be individualized, particularly for yellow fever vaccination, where infection risk must be weighed against the increased probability of serious vaccine-associated adverse events with advancing age [8, 26]. Shared decision-making may help align recommendations with traveler preferences and improve adherence to preventive strategies [27, 28].

## Main vaccines in senior travelers

The main vaccines used in senior travelers can be grouped into routine vaccines, travel-specific vaccines, recently introduced vaccines for which clinical experience remains more limited, and yellow fever vaccine as a distinct special scenario because of its specific risk-benefit considerations in older adults. Table 1 summarizes the main practical considerations.

**Table 1:** Main vaccines for senior travelers and key considerations for pre-travel vaccination

Vaccine type	Considerations
Routine vaccines	
Influenza	• Priority in travelers aged 65 years and older • Consider season, transit, airports, aircraft, cruises, and crowding • Enhanced formulations may be particularly relevant in frailty, chronic cardiopulmonary disease, or periods of increased circulation
COVID-19	• Review primary series and booster status • Consider comorbidity burden and immune dysfunction • Border or mobility-related requirements may be relevant
Pneumococcal	• Check documented previous doses • Verify whether earlier schedules were completed • Distinguish between vaccine-naïve, previously vaccinated, and incompletely vaccinated adults
RSV	• Particularly relevant in older adults with frailty or chronic conditions
Herpes zoster	• Consider because risk increases with age • Recombinant zoster vaccine provides high and persistent protection
Tetanus, diphtheria and pertussis	• Review booster status actively • Protection, particularly against diphtheria, may be inadequate in later life
Quadrivalent meningococcal conjugate	• Mainly relevant when itinerary or travel purpose creates a specific exposure or regulatory requirement • Especially relevant for Hajj or Umrah
**Travel-specific vaccines**	
Hepatitis A	• Do not assume protection, verify proactively • Give first dose as soon as travel is considered • If departure is imminent, immune globulin may need to be considered in selected groups
Hepatitis B	• Vaccine immunogenicity declines with age • Review vaccination history carefully rather than only counting administered doses • Low traveler uptake and short pre-travel timing may limit standard schedules
Typhoid	• Particularly relevant for higher-risk destinations, especially South Asia • Evidence directly focused on senior travelers remains limited
Rabies	• Review early if animal contact, prolonged stay, or limited access to post-exposure prophylaxis is expected • Access to rabies immune globulin may be poor in many settings • Standard multi-dose pre-exposure schedules appear more reliable in older adults
Japanese encephalitis	• Exposure-driven indication • Relevant for endemic areas with meaningful mosquito exposure • Evidence in adults older than 65 years is very limited
Tick-borne encephalitis	• Exposure-driven indication • More relevant with woodland exposure, outdoor activities, or repeated tick contact in endemic areas • Faster serodecline and lower neutralizing antibody levels may occur in older adults
Cholera	• Use selectively • Mainly for areas with active transmission and limited access to safe food, water, or timely care • No efficacy or safety data in patients aged over 65 years
**Recently introduced or special scenario vaccines**	
Yellow fever	• Special risk-benefit scenario in older travelers • Assess actual exposure risk, itinerary, season, planned activities, duration, and contraindications • Serious adverse events increase with age, especially from 60 years onward
Dengue	• Not a routine pre-travel tool in senior travelers • Use should be individualized and exposure-driven • Formal data in adults older than 60 years are lacking
Chikungunya	• Not a routine pre-travel tool in senior travelers • Use should be individualized and exposure-driven • Safety uncertainty is particularly relevant in older adults with comorbidities

### Routine vaccines relevant to travel

Reviewing routine vaccination is a core component of pre-travel care in all travelers. In older adults, reassessment of routine vaccines is particularly relevant because common vaccine-preventable infections may have more severe outcomes, protection may be less predictable, and travel may increase exposure through transit, crowding, seasonal mismatch, and itinerary-specific circumstances. Routine vaccines should therefore be reviewed according to vaccination history, clinical risk, and travel characteristics [29].

Influenza vaccination should remain a pre-travel priority in adults aged 65 years and older. A systematic literature review found that influenza in adults aged ≥ 65 years is associated with a high burden of hospitalization, ICU admission, and mortality, with comorbidities further increasing hospitalization, emergency department use, ICU admission, and mortality in this age group [30]. Protection may be lower and less durable later in life, especially during A(H3N2)-dominant or antigenically mismatched seasons [8, 30–33]. In seasons between 2012 and 2020, estimated influenza vaccine effectiveness among adults aged ≥ 65 years ranged from 12% to 66%, illustrating the variability of protection in this age group [30]. Compared with standard-dose vaccines, enhanced formulations have shown better protection against influenza-related outcomes and may be particularly relevant in senior travelers with chronic cardiopulmonary disease [31, 34], marked frailty [35], or travel during periods of increased circulation [31, 33].

COVID-19 vaccination also remains relevant in this population, as advanced age is associated with a substantially increased risk of severe disease, hospitalization, and death after SARS-CoV-2 infection, which is further influenced by immune dysfunction and the comorbidity burden [13, 36, 37]. Its travel relevance has also included links to mobility and border requirements [38, 39]. Available data show favorable safety and relevant immunogenicity in older adults for mRNA-1273 [37, 40], the chimpanzee adenoviral-vectored vaccine ChAdOx1 nCoV-19 [13], and MVC-COV1901 [41], while a booster strategy may also influence the immune response in this age group [13, 37]. In a systematic review and meta-analysis including 3,404,696 adults aged > 60 years, COVID-19 vaccines reduced SARS-CoV-2 infection (OR = 0.38; 95% CI, 0.23–0.65; *p* = 0.0004) and COVID-19-related mortality (OR = 0.16; 95% CI, 0.10–0.25; *p* < 0.00001) in older adults. Antibody seroconversion and geometric mean titres also increased significantly after vaccination in this population (OR = 24.42; 95% CI, 19.29–30.92; and SMD = 0.92; 95% CI, 0.64–1.20, respectively) [36].

*Pneumococcal* vaccination is increasingly relevant because community-acquired pneumonia contributes substantially to morbidity later in life [23, 42, 43]. In the CAPiTA randomized trial involving 84,496 adults aged ≥ 65 years, PCV13 reduced first episodes of vaccine-type pneumococcal community-acquired pneumonia by 45.6% (95.2% CI, 21.8–62.5), nonbacteremic and noninvasive vaccine-type community-acquired pneumonia by 45.0% (95.2% CI, 14.2–65.3), and vaccine-type invasive pneumococcal disease by 75.0% (95% CI, 41.4–90.8) [44]. Newer conjugate vaccines have expanded serotype coverage: PCV15 includes two additional serotypes beyond PCV13, whereas PCV20 includes seven additional serotypes [45]. PCV15 has shown superior immunogenicity compared with PCV13 (GMT ratio 1.11; 95% CI, 1.02–1.20), while PCV20 met noninferiority criteria versus PCV13 at 1 month after vaccination (GMT ratio 0.84; 95% CI, 0.81–0.87) [45]. In practice, decisions should be guided by documented previous doses and whether earlier schedules were completed, since current recommendations distinguish between vaccine-naïve adults, previously vaccinated individuals, and those with incomplete schedules [46, 47].

Pre-travel consultation may also provide an opportunity to update other age-relevant vaccines. RSV vaccination is particularly relevant in older adults with frailty or chronic conditions. RSV vaccination reduced RSV-related lower respiratory tract infection/disease, with an overall vaccine efficacy of 61% in adults older than 60 years. Efficacy was 73% when the analysis was restricted to prefusion vaccines, and one trial reported 94.1% efficacy against severe RSV-related lower respiratory tract infection/disease [48]. The recombinant zoster vaccine offers high and persistent protection. In a systematic review and meta-analysis, vaccine efficacy against herpes zoster was 94.0%, in adults aged ≥ 50 years, and 91.3% in adults aged ≥ 70 years [49]. Booster vaccination against tetanus, diphtheria and pertussis should be reviewed actively, as protection may be inadequate later in life [50]. This is particularly relevant for diphtheria, since a substantial proportion of adults, especially in older age groups, lack protective antibody concentrations, whereas tetanus-specific antibody concentrations are generally higher [51]. Pertussis protection is also suboptimal in adults, and severe pertussis appears to be more frequent in older adults, with a study reporting a 5-fold higher hospitalization risk in patients aged 65–74 years and a 9-fold higher risk in those aged > 75 years compared with adults aged 45–54 years [52]. A recent registry analysis also illustrates the implementation gap: only 30.2% of adults aged ≥ 50 years were up to date for tetanus vaccination, 30.1% for diphtheria, and 20.8% for pertussis vaccination, with pertussis coverage declining to 13.5% among those aged ≥ 85 years [50]. Quadrivalent meningococcal conjugate vaccination becomes mainly relevant when itinerary or travel purposes create a specific exposure or regulatory need, especially for Hajj or Umrah [53, 54].

### Travel-specific vaccines

Travel-specific vaccination should be guided by exposure risk rather than by age alone. However, age remains clinically relevant because older adults may experience more severe outcomes from some travel-associated infections and may show reduced vaccine responsiveness [29]. Assessment should therefore establish not only whether a vaccine is indicated, but also whether timing, prior immunity, coexisting conditions, expected responsiveness, and access to post-exposure care make vaccination particularly important or of limited practical value [29].

Hepatitis A remains relevant in senior travelers because disease severity increases with age and may be further amplified by chronic liver disease or other comorbidities [55, 56]. This remains clinically relevant because travel-associated hepatitis A continues to occur despite vaccine availability. In an analysis of 254 international travelers with acute hepatitis A, 53 of 54 travelers with available vaccination data (98%) were unvaccinated and 59% of those with clinical information were hospitalized [57]. Among U.S. adults aged 18–49 years who had traveled to countries of high or intermediate hepatitis A endemicity, only 26.6% reported ≥ 1 hepatitis A vaccine dose and 16.9% had completed a ≥ 2-dose series [58]. In older travelers, the need for pre-travel evaluation is reinforced by age-related differences in both baseline immunity and vaccine response. In a study of 427 international travelers aged > 40 years traveling to endemic areas, the overall prevalence of hepatitis A antibodies was 78.9%, increasing with age from 62.6% in those aged 40–49 years to 91.7% in those aged 60–69 years and 97.5% in those aged 70–95 years. These findings support screening for hepatitis A antibodies before selective vaccination in older travelers when time before departure allows it, particularly because many may have naturally acquired immunity [59]. Nevertheless, vaccination should be planned early in susceptible older travelers, as available evidence suggests reduced seroprotection after a single primary hepatitis A vaccine dose in adults aged > 50 years, with improved protection after booster vaccination [19]. Timing is also important since the first dose should be given as soon as travel is considered, and, according to some guidelines, selected groups may warrant immune globulin when departure is imminent [56, 60]. Possible coadministration effects should also be considered, as not all combinations appear immunologically neutral [61, 62].

For hepatitis B virus (HBV), the main age-related issue is declining vaccine immunogenicity, which makes protection less reliable in later life than in younger adults [63–65]. Although travel-related HBV exposure is less common than HAV exposure is, infection may become chronic and progress to cirrhosis, so failure to achieve protection may have greater consequences in older travelers [64, 66]. Poor responses after combined hepatitis A/B vaccination and the benefit of additional or reinforced doses support careful review of vaccination history and likely protection [64, 67], particularly because traveler uptake remains suboptimal and some present too close to departure to complete standard schedules comfortably [68, 69].

The relevance of typhoid vaccination is greatest for higher-risk destinations, particularly South Asia, where mortality is higher in older adults than in younger patients [29, 70, 71]. Current options include the Vi polysaccharide vaccine and Ty21a, while conjugate vaccines may induce stronger and longer-lasting immune responses, although evidence directly focused on senior travelers remains limited [72–74]. In response to the increasing spread of resistant Salmonella strains, WHO recommends the implementation of typhoid vaccination as a strategy to reduce disease burden and antibiotic use, despite the limited immunogenicity of some available vaccines [75].

Within this exposure-based framework, rabies should be assessed early, as its relevance depends on likely animal contact and access to reliable post-exposure prophylaxis. This is particularly important because rabies is almost invariably fatal once symptoms appear [29]. Category III exposures include transdermal bites or scratches, contamination of mucous membranes with saliva, or licks on broken skin, and generally require rabies immune globulin in addition to vaccination in previously unvaccinated travelers [76]. However, access to rabies immune globulin may be limited during travel: studies in international travelers indicate that only 5–20% received rabies immune globulin in the country of exposure when indicated, and a multicenter survey found that approximately two-thirds of travelers with an indication for rabies immune globulin who started post-exposure prophylaxis during travel did not receive it [76]. Available evidence suggests that standard multi-dose pre-exposure schedules provide more reliable and durable immunity than reduced schedules do in older adults, while booster responses remain strong even years later [9, 77]. Therefore, rabies risk should be reviewed early, particularly when travel likely involves animal contact, prolonged stays, or destinations with limited access to reliable post-exposure prophylaxis [29].

The same anticipatory approach should guide the use of other destination-dependent vaccines, which should be selected according to the likelihood of exposure. In this context, Japanese encephalitis becomes relevant when itineraries involve endemic areas with meaningful mosquito exposure, although studies in adults older than 65 years remain very limited [78–80]. Tick-borne encephalitis deserves particular attention later in life because poor neurologic outcomes are more frequent with increasing age, and older adults may show lower neutralizing antibody levels and faster serodecline despite high initial seropositivity after primary vaccination [81, 82]. Cholera vaccination should remain selective, mainly for travelers going to areas with active transmission where safe food, water, or timely medical care may be difficult to secure, especially if dehydration is poorly tolerated because of comorbidity, although no efficacy or safety data are available for patients aged over 65 years [83].

### Yellow fever vaccination as a special scenario

Yellow fever vaccination in older travelers represents a distinct clinical scenario. It is not a routine age-based intervention but an itinerary-dependent decision relevant when travel involves endemic areas or countries requiring proof of vaccination, and when exposure is credible rather than merely theoretical [84]. Assessment should move beyond destination labels alone and consider route, season, planned activities, travel duration, local virus transmission, actual mosquito exposure, and the risk of adverse events following immunization [15, 84, 85]. Quantitative risk estimates may help frame this decision. For an unvaccinated traveler spending 2 weeks in an endemic area, the estimated risk of yellow fever illness has been reported as approximately 50 cases per 100,000 travelers in West Africa and 5 cases per 100,000 travelers in South America, with estimated mortality risks of 10 and 1 per 100,000 travelers, respectively [86]. Although travel-associated cases are rare, outcomes can be severe: among 11 yellow fever cases reported in travelers from the U.S. and Europe between 1970 and 2015, 8 were fatal [86].

Advanced age is universally classified as a precaution because immune aging may be associated with a delayed humoral response and prolonged vaccine viremia [85]. Pre-travel decision-making should therefore weigh the likelihood of true exposure against vaccine-related risk, particularly in first-time vaccine recipients and in older adults with relevant comorbidities or contraindications [15]. In practice, the threshold for vaccination should be higher in older adults but not indiscriminate [84]. Certain coexisting conditions, particularly specific immunocompromised states, thymic disease or thymectomy, have moved the discussion from precaution to true contraindication, in which a live yellow fever vaccine may pose unacceptable risk [84, 87].

Serious adverse events after yellow fever vaccination increase with age, especially from 60 years of age onward [88–90]. The most clinically relevant syndromes are yellow fever vaccine-associated neurologic disease and viscerotropic disease, which occur almost exclusively in first-time vaccine recipients [84, 85]. A 2022 meta-analysis revealed that the risk of serious adverse events was approximately threefold higher in those aged 60 years and could reach fivefold higher in those aged 70 years or older, while the relative risk of vaccine-associated viscerotropic disease was 6.59 in the elderly population [91]. Moreover, U.S. surveillance from 2007 to 2013 reported serious adverse event rates after yellow fever vaccination of 6.5 per 100,000 doses in persons aged 60–69 years and 10.3 per 100,000 doses in those aged ≥ 70 years, with overall reporting rates of 0.8 per 100,000 doses for neurologic disease and 0.3 per 100,000 doses for viscerotropic disease [92]. Viscerotropic disease is rare but severe, with an estimated case fatality of approximately 65% [93]. Vaccine hesitancy is also relevant, as more than 36% of seniors planning travel to endemic regions reportedly refuse vaccination after learning of the elevated adverse-effect risk [94].

Yellow fever vaccine should therefore be considered only when the itinerary entails significant and unavoidable exposure risk [84]. When travel to high-risk endemic areas in South America or Africa is inevitable, the risk of wild-type infection may outweigh the statistical risk of severe vaccine-associated adverse events [84, 85]. Conversely, vaccination should be avoided when exposure risk is negligible or absent, or when relevant contraindications are present, particularly immunocompromised states or thymic disease [84, 85, 95].

### Recently introduced or emerging vaccines

Recently introduced arboviral vaccines are increasingly relevant in older travelers because both dengue and chikungunya can cause substantial morbidity in this population, whereas age narrows the margin for uncertainty regarding safety, durability of protection, and real-world effectiveness. At present, dengue and chikungunya vaccines should be considered selectively in senior travelers, taking into account anticipated exposure, comorbidities, and remaining evidence gaps [96].

Dengue vaccination is currently more immediately relevant in travel medicine than chikungunya vaccination because dengue affects travelers more frequently and may lead to substantial morbidity and non-negligible hospitalization rates [96]. In this setting, TAK-003 is the main relevant option, but it should be discussed as a live-attenuated tetravalent dengue vaccine, and the usual contraindications and precautions for live vaccines apply [96]. It showed 81.1% efficacy against all serotypes in seropositive individuals and reduced hospitalization by 97.2%, with protection beginning 14 days after the first dose [96, 97]. However, the benefit in seronegative travelers, particularly against DENV3 and DENV4, is uncertain or absent in some analyses [96–98]. This uncertainty is clinically relevant because antibody-dependent enhancement is a recognized concern in dengue vaccination: subneutralizing or imbalanced antibody responses may theoretically increase the risk of more severe disease after subsequent infection with a heterologous serotype [99]. Although TAK-003 has not shown the same safety signal that led to restrictions for CYD-TDV in seronegative individuals [96], WHO indicates that vaccine-induced disease enhancement cannot be fully excluded in seronegative recipients for DENV-3 and DENV-4 [100]. Importantly, data in adults older than 60 years are lacking [96, 97, 101].

Chikungunya vaccination is also relevant in older travelers because older age and comorbidities may increase the clinical consequences of acute infection, while persistent arthralgia or arthritis can result in prolonged functional impairment [102, 103]. The live-attenuated vaccine showed strong single-dose immunogenicity in adults, including older adults, but post-licensure surveillance revealed serious neurological or cardiac adverse events concentrated in older adults with comorbidities, prompting precautionary responses [102–104]. In contrast, the recombinant virus-like particle chikungunya vaccine is now an authorized non-live, nonreplicating option in some regions, including the U.S. and the European Union [105]. Phase 3 data in adults aged 65 years and older showed favorable seroresponse rates and no vaccine-related serious adverse events or deaths, although follow-up remains limited [106].

Overall, dengue and chikungunya vaccines should currently be considered selectively in senior travelers, particularly when anticipated exposure justifies their use despite remaining age-specific evidence gaps [96, 102, 104].

## Prevention beyond vaccination in senior travelers

### Food and water precautions

Traveler’s diarrhea remains a major travel-related problem, as gastrointestinal symptoms and acute non-specific diarrhea are the leading causes of travel-related medical consultation across age groups, including older adults [3]. Its overall incidence has been estimated to be 20% to 50% among travelers [24]. However, traveler’s diarrhea appears to be less common in older adults than in younger travelers, possibly because of differences in dietary habits, lower exposure to risk, or variation in the gut microbiota composition [3]. This lower incidence has also been reported in longer stays in high-risk settings, where older travelers may maintain better food hygiene practices or undertake different types of journeys than younger individuals do [3]. Consistent with this, older age has been significantly associated with better adherence to food and water precautions [107]. In contrast, younger travelers, especially those aged 18 to 35 years, are less likely to follow low-risk behaviors and are more likely to consume raw meat or fish, have contact with fresh water, neglect handwashing, fail to avoid salads, or eat without utensils [108]. Despite the wide availability of information on safe food handling and treated water use, adherence remains limited overall [109], although relatively few studies have specifically assessed compliance with food and water recommendations during travel. In a survey of 644 travelers, 388 respondents reported consuming street food, 245 ingested ice cubes in drinks, and 116 shared meals with local residents during travel, whereas only 169 reported using antibacterial gel [109]. Adherence to food recommendations appears particularly low among travelers visiting friends and relatives, with one study reporting full adherence in only 6% of adults visiting friends and relatives [110]. Compliance also tends to decline at both extremes of travel duration and decreases further for travelers staying abroad for more than six months [108]. In older adults and travelers with chronic disease, diarrheal illness may also lead more readily to clinically relevant complications, particularly dehydration, and food and water precautions do not fully prevent illness [24, 107]. For this reason, senior travelers should receive practical advice on episode management, whereas antibiotic treatment should be reserved for severe diarrhea or dysentery and used cautiously in view of multimorbidity and the risk of resistant intestinal colonization [8, 107].

### Vector avoidance measures

Vector avoidance measures are a general principle of travel medicine and are not specific to older travelers [8]. Nevertheless, they remain an important component of prevention in this population, particularly in travelers who receive selected vaccines against vector-borne infections, since those vaccinated against diseases such as Japanese encephalitis or chikungunya may still remain at risk of other mosquito-borne infections, including malaria and dengue [79]. This is particularly important because some pre-travel consultations continue to prioritize vaccines while paying insufficient attention to basic bite prevention [10, 15].

### Respiratory and hygiene measures

Respiratory infections are a frequent cause of travel-related illness, particularly in crowded settings and mass gatherings, where exposure may begin during transport and transit through buses, trains, and airports [111]. Among ill pilgrims attending Hajj, respiratory tract infections have accounted for up to 60% of medical complaints [111]. The transmission of respiratory viruses, including seasonal influenza, occurs through aerosols, droplets, and contaminated surfaces, and prevention is complicated by the fact that up to 40% of influenza cases may be transmitted before symptom onset [111]. Although respiratory and hygiene measures are not specific to older adults, respiratory prevention deserves particular emphasis in senior travelers because respiratory tract infections are common during travel in older adults and may be associated with greater clinical impact in this population. In an analysis of 7,034 ill travelers aged ≥ 60 years, respiratory disease accounted for 14.6 cases per 100 ill travelers, compared with 10.3 per 100 among travelers aged 18–45 years (*p* < 0.001). Lower respiratory tract infections, including pneumonia and bronchitis, were also proportionally more frequent in older travelers than in younger adults (7.3 vs. 2.8 cases per 100 ill travelers; *p* < 0.001) [112]. In this context, practical hygiene measures remain relevant, including ventilation of crowded environments, hand disinfection, and handwashing after contact with potentially contaminated surfaces [111]. Evidence suggests that face masks and careful hand hygiene may reduce transmission when applied early and consistently, although their effectiveness depends heavily on adherence and correct use [111].

### Education, adherence, and risk perception in experienced travelers

In experienced travelers, adherence is shaped not only by access to recommendations but also by risk perception, as many report a low perceived risk of infection and rely on previous travel history, assuming that they already understand the relevant risks or that the destination poses no meaningful threat [108, 109]. As a result, more frequent international travelers may be less likely to attend specialized pre-travel consultations than those who travel less often, despite the fact that this confidence may coexist with important knowledge gaps, including failure to recognize that a destination is highly endemic for infections such as malaria [108, 109]. Within the older population, however, this relationship appears more nuanced. Some evidence suggests that older adults may seek pre-travel medical advice less frequently than younger travelers do, possibly because of confidence derived from a lifetime of prior travel experiences [3]. Nevertheless, once advice is received, older age has been associated with better adherence to preventive behavioral measures [3, 107]. Pre-travel counseling in this group should also include sexually transmitted infections, even if this topic is often less emphasized in older travelers, because they are not exempt from sexual risk behaviors during international travel [108, 109]. The travel setting may favor anonymity, distance from home, and departure from usual norms, which can facilitate casual sexual encounters across age groups, underscoring the need to address safer sexual practices explicitly as part of travel health advice [109].

**Fig. 2:**
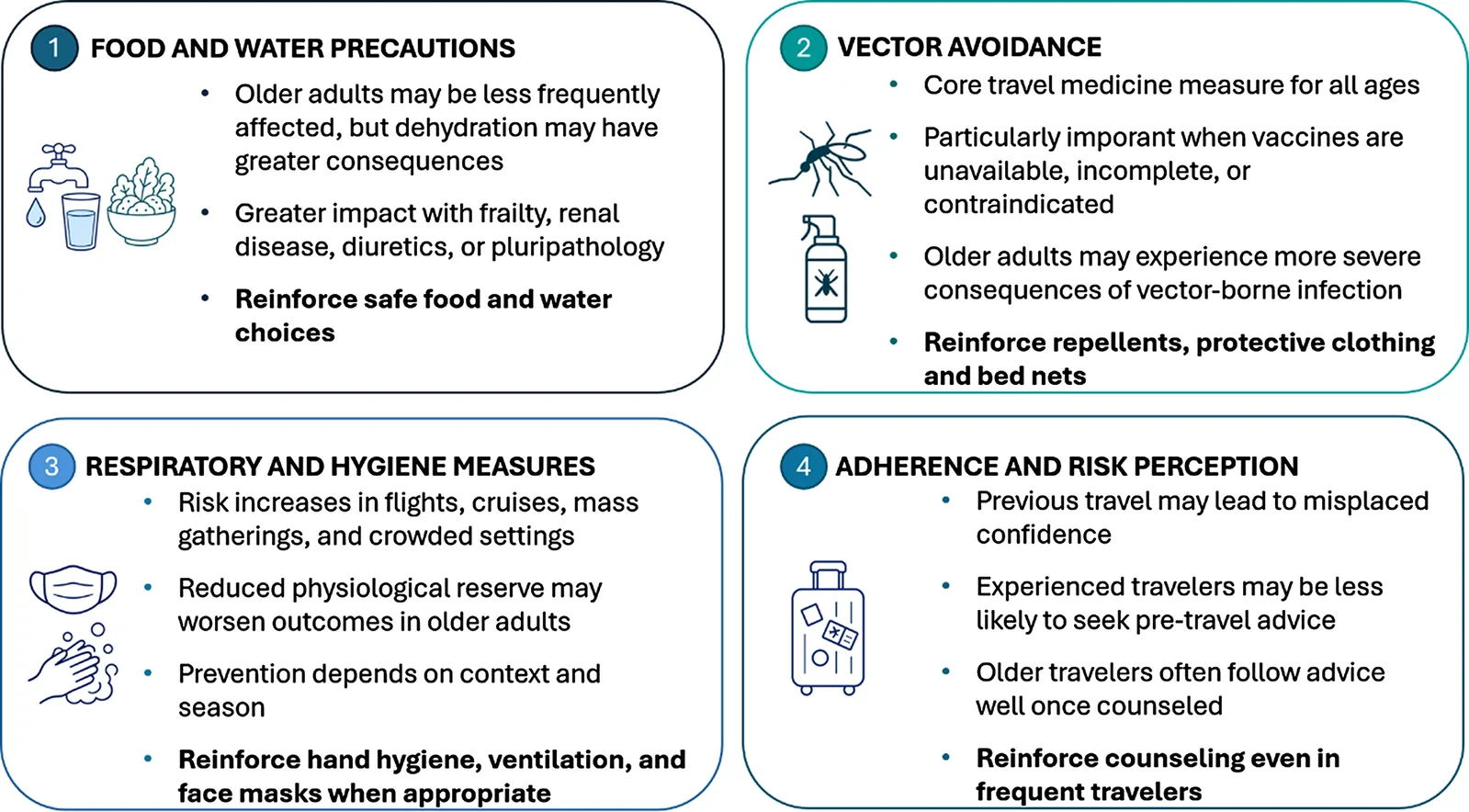
Key non-vaccine preventive measures and behavioral factors relevant to senior travelers during pre-travel counseling

A concise summary of general preventive measures beyond vaccination is presented in Fig. 2.

## Challenges and knowledge gaps

The main limitation of the current evidence base is not the absence of data altogether, but the limited applicability of much of the available evidence to the clinical reality of senior travelers. Important uncertainties persist regarding vaccine immunogenicity, safety, durability of protection, and optimal implementation in older adults. As a result, recommendations in this population still rely too often on extrapolation, limited direct evidence, or expert opinion.

### Limited age-specific evidence and underrepresentation of senior travelers

A major challenge in travel vaccination for older adults is the limited clinical granularity of the available evidence. Many vaccine studies rely on chronological age categories, often using cut-offs such as 60 or 65 years, but provide limited stratification by frailty, comorbidity burden, functional status, prior immunity, or travel-related exposure [113]. As a result, findings may be difficult to translate into individualized pre-travel decisions, particularly because chronological age alone does not distinguish between healthy, active senior travelers and older adults with substantial frailty or multimorbidity [29, 113]. Part of the available evidence on vaccine coadministration also derives mainly from pediatric studies, leaving an important gap in adults and older populations [7].

These limitations are evident across several vaccines relevant to travel medicine. For dengue, pivotal studies of TAK-003 included participants aged 4 to 60 years only, leaving no direct evidence in adults older than 60 years [114]. A similar limitation applies to yellow fever vaccine, for which data on immunogenicity in adults aged 60 years or older remain scarce, particularly regarding the durability of protection after a single dose [115]. In rabies prevention, evidence on standard and accelerated intradermal schedules in adults older than 50 years remains limited, as does evidence on booster responses after exposure [9]. Comparable gaps are also observed for Japanese encephalitis and chikungunya vaccines [11, 12, 78].

An additional challenge is that senior travelers are not necessarily representative of the general older population. On one hand, highly selected vaccine trials may exclude older adults with relevant frailty or uncontrolled comorbidities, thereby limiting applicability to clinically vulnerable travelers. This was explicitly observed in the ChAdOx1 nCoV-19 trials, where adults aged 65 years or older were excluded if they had a Dalhousie Clinical Frailty Scale score of 4 or higher, or severe or uncontrolled medical comorbidities [13]. On the other hand, data from general older populations may not reflect the generally healthier and more mobile adults who attend pre-travel clinics [4]. The lack of standardized frailty definitions, limited reporting of functional status, and potential selection bias in observational studies further reduce the ability to translate available evidence into travel-specific recommendations [4, 29]. Future studies and observational travel-medicine cohorts should therefore include adults aged 65 years or older and report clinically meaningful variables such as frailty, comorbidity burden, functional status, prior immunity, travel itinerary, and exposure profile, rather than relying on age alone.

### Uncertainty in immunogenicity and duration of protection

Beyond the limited availability of age-specific studies, an additional challenge in senior travelers is the uncertainty surrounding both the magnitude and persistence of vaccine-induced protection. In this population, protection may be less predictable and less durable than in younger adults, making assumptions based on standard schedules or remote vaccination history less reliable [3, 7]. This issue is relevant not only for recently introduced vaccines but also for long-established vaccines commonly considered in pre-travel care [3, 8]. An additional limitation is that many studies and clinical assessments rely primarily on antibody titres as markers of protection [18, 113]. These endpoints may not fully capture memory B-cell responses, T-cell-mediated immunity, or boosterability, which are particularly relevant when interpreting apparent waning immunity in older adults [18].

This uncertainty is particularly evident for yellow fever, Japanese encephalitis, and rabies. For yellow fever, the assumption of lifelong protection after a single dose remains uncertain in older adults, although a 10-year follow-up showed sustained seropositivity in 100% of participants (36/36), with comparable neutralizing antibody concentrations versus younger controls (6.7 vs. 8.6 IU/mL, *p* = 0.5) [115]. For Japanese encephalitis, available data suggest waning seroprotection in later life and limited evidence to guide booster strategies [80]. In persons over 65 years, seroprotection decreased after a two-dose primary series of the Vero cell-derived inactivated vaccine [79], while with the live recombinant chimeric vaccine, seropositivity declined from 100% at 2 to 5 years to 92.9% (52/56) beyond 5 years [78]. Rabies presents similar challenges, as older age has been associated with weaker and less durable responses after pre-exposure prophylaxis. Seropositivity after a primary intradermal course was 89.4% in individuals over 50 years [9], and long-term seroprotection was lower in those aged ≥ 50 years than in those < 50 years (75.0% vs. 88.2%) [11].

Comparable uncertainties also persist for newer arboviral vaccines, since adults older than 60 years were not included in pivotal dengue studies [114], and for chikungunya the durability of protection and need for boosters remain unresolved [12]. In addition, age-related reductions in immunogenicity are relevant for hepatitis A, hepatitis B, and influenza. As stated before, reported seroprotection was lower in older adults for hepatitis A after the first dose (65% vs. 100%) and hepatitis B (64.8% in ≥ 65 years vs. 98.6% in 20–24 years) [8]. For influenza, reduced responses have also been reported, with H5N1 seroprotection at day 42 of 74% in subjects > 65 years versus 91% in younger adults, and H1N1 seroconversion of 23% in adults aged 61–86 years versus 54% in those < 60 years [8, 111, 116].

### Need for individualized vaccination strategies

These evidence gaps support individualized rather than standardized risk stratification in older travelers [3, 8, 87]. Vaccine decisions in travelers aged 60 years or older should therefore be made case by case, integrating medical background, destination, itinerary, exposure profile, likely vaccine responsiveness, and the balance between expected benefit and potential risk [3, 87].

This is particularly important when the balance between benefit and risk is uncertain, especially for live-attenuated vaccines or in the setting of immunocompromise [8, 15]. Yellow fever vaccination remains the clearest example, as medical exemption may be appropriate when vaccine-related risk outweighs expected benefit in adults older than 60 years [3, 8]. Similar case-by-case judgement is required for rabies pre-exposure prophylaxis [8] and for travelers with unclear vaccination histories, in whom selective serology may help avoid both underprotection and unnecessary vaccination [22].

## Conclusions

Vaccination remains a key component of pre-travel prevention in senior travelers, but it should be framed within an individualized assessment rather than based on chronological age alone. Expected benefit varies according to health status, prior immunity, and itinerary. Routine vaccines should be reviewed systematically, whereas travel-specific vaccines should be guided by destination, itinerary, timing before departure, and risk-benefit balance, particularly for yellow fever vaccination. Vaccination should also be integrated with non-vaccine preventive measures. Despite growing evidence, important uncertainties remain because age-specific data are limited and frail older adults remain underrepresented in clinical studies.

## Data Availability

No datasets were generated or analysed during the current study.
